# Emerging practices of healthcare waste management among private surgeries: A case of the City of Tshwane

**DOI:** 10.4102/hsag.v30i0.2845

**Published:** 2025-03-18

**Authors:** Tshepiso K. Hlako, Thabiso J. Morodi, Matodzi M. Mokoena, Gomotsegang F. Molelekwa

**Affiliations:** 1 Department of Environmental Health, Faculty of Science, Tshwane University of Technology, Pretoria, South Africa; 2 Regional Water and Environmental Sanitation Centre, Department of Civil Engineering, Kwame Nkrumah University of Science and Technology, Kumasi, Ghana

**Keywords:** healthcare waste management, healthcare waste norms and standards, private surgeries, healthcare waste storage, healthcare waste treatment, public health and healthcare waste management

## Abstract

**Background:**

This study presents the emerging practices of healthcare waste (HCW) management prevailing in most private surgeries within the City of Tshwane Metropolitan Municipality. This study examined the existing norms and standards of HCW management to unravel the minimum lawfully acceptable practices in South Africa and the world.

**Aim:**

The aim of this study was to assess the management of HCW in private surgeries within the City of Tshwane Metropolitan Municipality.

**Setting:**

The study was conducted in the City of Tshwane Metropolitan Municipality, Gauteng province, South Africa.

**Methods:**

A mixed research methodology was employed in collecting and analysing data collected from 109 professional doctors, dentists, nurses, administrative staff and cleaners working in private surgeries.

**Results:**

The findings revealed that private surgeries generated 98.17% of infectious waste, while 1.83% was general waste. Most containers adhered to South African National Standards guidelines, but knowledge gaps were found regarding treatment and disposal methods. A total of 92.7% of private surgeries used private companies for waste collection, transport and disposal. Those who used private waste management companies were given Waste Manifest documentation as proof of disposal at an approved facility.

**Conclusion:**

Private surgeries in the City of Tshwane Metropolitan Municipality managed hazardous HCW satisfactorily, but improvements in container usage and waste handling procedures are recommended.

**Contribution:**

The findings of the study can be used to develop comprehensive HCW management guidelines for private surgeries, to help them better manage the HCW they generate.

## Introduction

Improper management of healthcare waste (HCW) is associated with innumerable known acute and chronic public health risks (Health Professions Council of South Africa [HPCSA] 2016). A study by Diaz, Savage and Eggerth (2005) found that workers in healthcare settings often contract pathogenic infections because of their exposure to bodily fluids associated with malpractices in the handling of HCW. Management of HCW is increasingly becoming a global concern in both developed and developing countries (Hayleeyesus & Cherinete 2016). According to Machate et al. (2021), common factors that contribute to improper management of HCW include: (1) inconsistencies in the characterisation of HCW and its properties, (2) absence of universal definition of HCW and (3) lack of strict compliance enforcement by authorities. Diaz et al. (2008) add that effective HCW management has major financial implications, which is of particular concern in developing countries. The latter is anchored in the works of Caniato, Tudor and Vaccari (2015) and Chisholm et al. (2017) who found that developed countries are better able to manage HCW than their counterparts because of their financial muscles.

**FIGURE 1:**
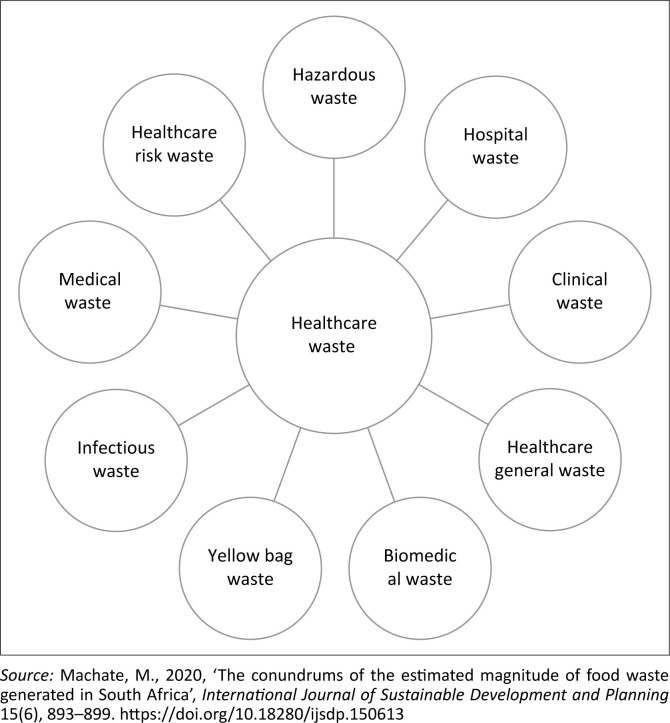
Concepts used interchangeably with ‘healthcare waste’.

On the second factor, it is worth noting that the World Health Organization (WHO 2014) defines HCW as waste that originates from a healthcare facility, health research facility, health-related laboratories and home healthcare activity (dialysis & insulin injections). The aforesaid is consistent with the definition from the *South African National Health Act of 2003* (Act No 61 of 2003). Machate et al. (2021) identify numerous concepts that are interchangeably used to refer to HCW (Figure 1).

Bendjoudi et al. (2009) add to the argument that inconsistency in what is defined or characterised as HCW contributes to its improper handling. A study by Cook et al. (2023) concurs that there is no universally accepted definition of HCW. Furthermore, Diaz et al. (2008) aver that without well-defined terms, major difficulties and misunderstandings are bound to occur. Broadly, the WHO (1999) categorised HCW into: (1) non-hazardous (general or non-infectious) and (2) hazardous waste (infectious). These broad categories are, according to Mohseni-Bandpei et al. (2019) based on the HCW’s potentially infectious nature and toxicity of the materials or component, which according to WHO (1999) is estimated at 85% (non-hazardous), and only 15% is hazardous (ed. Chartier 2014; eds. Prüss, Giroult & Rushbrook 2014; WHO 2014).

**FIGURE 2:**
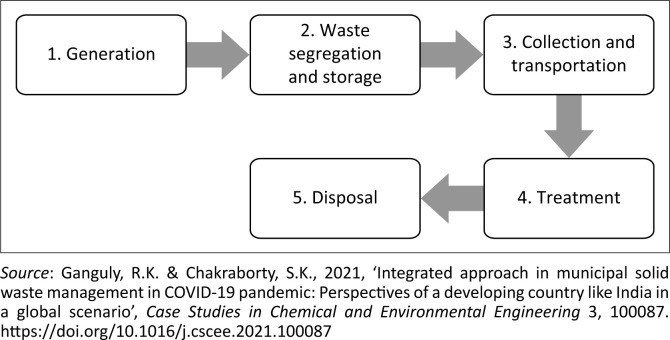
Fundamental elements of healthcare waste management.

According to Das et al. (2021), Ganguly and Chakraborty (2021) and Fadaei (2023), fundamental elements of HCW management consist of the following steps (Figure 2).

**FIGURE 3:**
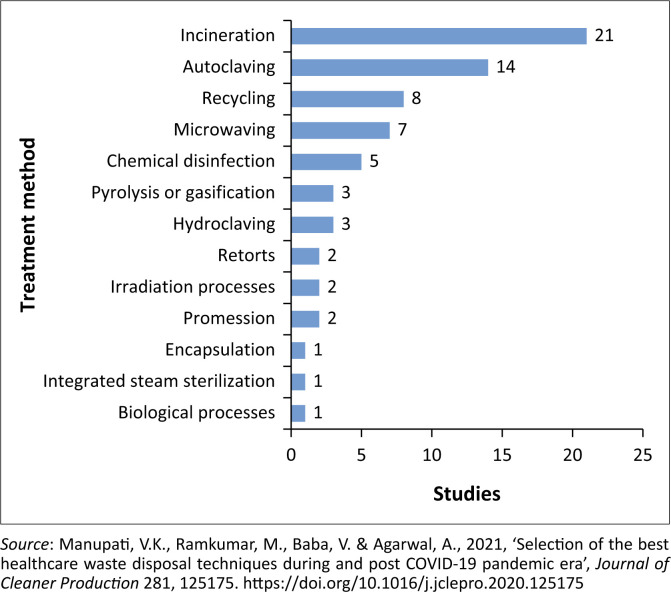
Frequency of studies that identified each treatment method.

The norms and standards of handling HCW from generation to final disposal are regulated in South Africa, in line with the WHO (2017). The South African Bureau of Standards (SABS 2008) presents a peremptory colour coding systems that must be used for HCW containers, as part of the minimum compliance requirement in the management of HCW. It is evident that proper segregation and colour coding of HCW containers help to prevent and minimise the mixing of hazardous HCW with non-hazardous HCW, which may lead to the non-hazardous HCW stream being contaminated. The prevention of non-hazardous HCW from cross-contamination by hazardous HCW is essential in reducing the potential for this waste to be hazardous. Manupati et al. (2021) explored alternative practices used for the treatment and disposal of HCW (Figure 3).

A study in Botswana by Mmereki et al. (2017) found that common methods for the treatment of hazardous HCW included incineration and autoclaving. A study by Machete and Shale (2015) highlights the fact that the hazardous waste from HCW belongs to nine classes of hazardous substances, regulated by the *Hazardous Substances Act*. Furthermore, the study points out that HCW falls within the four hazard rating matrices. The class of landfill where such HCW can be disposed of is ultimately determined by their hazard rating. It is thus necessary for HCW to be treated to reduce their hazard rating before final disposal. Thus, the current study examined the prevailing practices of HCW management used by private surgeries of the City of Tshwane Metropolitan Municipality Area, in comparison to international and national minimum acceptable HCW management norms and standards.

## Research methods and design

### Study design and site

The study was conducted in the City of Tshwane Metropolitan Municipality, located in Gauteng province, South Africa. A mixed method approach was adopted in this study which assessed the management of HCW in private surgeries in the City of Tshwane Metropolitan Municipality as defined in the works of Trochim and Donelly (2001), and Ubisi, Khumalo and Nealer (2019). The approach incorporated both descriptive and inferential statistical analyses of the collected data, along with various presentation techniques that combined qualitative and quantitative figures, as well as other methods, to present the study results (Creswell & Creswell 2017).

### Study population and sampling strategy

The study population included medical doctors, administrators, nurses and cleaners at private surgeries who are working 15 days or more per month. Medical doctors who do locums (relievers or part time) were excluded from participating in the study. Convenience sampling method was used to select the required number of private surgeries following a sample size that was determined using the Slovin formula (Equation 1):


$$N=\frac{N}{1=NE}=139$$ [Eqn 1]

where *N* is the population, and E represents the error of estimation (Hotjar 2021).

At the time of conducting the study, the population of private surgeries was 213, and the sample size obtained through the Slovin formula was 139 private surgeries.

### Data collection

A structured questionnaire containing grouped questions was used to collect quantitative data, whereas qualitative data were obtained through observations. The questionnaire and observations focused on the generation of HCW specifically on the type and quantity of waste. Furthermore, they focused on HCW storage regarding the type of containers used, the type of the storage facility and cleanliness of the storage facility. In addition, observations included checking the labelling, liners and lids of the container used, whether the container was damaged, properly placed and any signs of spillage. Other areas of focus were segregation, transportation, treatment and disposal, respectively. The researcher was taking down notes during the observation exercise, which were used when doing data analysis. Data were collected between March 2022 and April 2022. Because of coronavirus disease 2019 (COVID-19) restrictions, appointments were made telephonically with the study participants. The researcher and the study participants maintained social distancing and put on a face mask during the appointment. The researcher filled in the questionnaires as the study participants were responding to the questions.

### Data analysis

This study collected both qualitative and quantitative (mixed) data sets. All collected data were firstly summarised and secondly categorised according to the characteristics and the frequencies related thereof. Collected data were transcribed and captured onto MS Excel spreadsheets. The Statistical Package for Social Sciences (SPSS version 28) was used for the analysis of both the qualitative and quantitative data. Furthermore, frequencies of different management practices (categories) were counted, and inferences were drawn from each data set. Numerical data were presented as graphs and tables (Burns & Grove 2009).

### Ethical considerations

The study acquired ethical approval from the Faculty committee for Research Ethics (FCRE) of the Tshwane University of Technology on 14 February 2022 (Reference no.: FCRE 2021/12/005 [SCI][FCPS02]). Permission to conduct the study was granted by the City Manager of the City of Tshwane Metropolitan Municipality.

Consent forms were issued to participants at private surgeries who were working 15 days or more per month. Participants who worked part time were excluded from this study. Participants were informed about the process of the research as well as voluntary participation. The participants were also informed that they could withdraw from participating in the study at any time if they were uncomfortable or did not want to participate in the study anymore. In addition, participants’ anonymity was ensured, only the data acquired were utilised, and no names were mentioned. The participants were treated with dignity and their autonomy was recognised. All parts of the study were conducted according to internationally accepted ethical principles.

## Results

**FIGURE 4:**
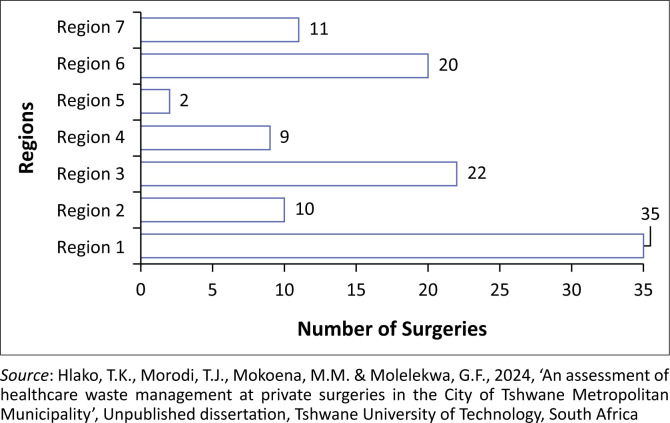
Number of participated private surgeries and their respective regions.

In this study, 55 (50.5%) males and 54 (49.5%) females were included, making a total of 109 participants. All participants were adults of 18 years and above as defined in section 17 of the *Children’s Act of 2005* (Act No. 38 of 2005). Figure 4 presents the number of private surgeries that participated in the study and their regions within the City of Tshwane Metropolitan Municipality.

The number of private surgeries that participated in the study per region ranged from 2 to 35, with Region 1 having the largest number of private surgeries that participated in the study, and Region 5 having the least number of participated private surgeries (Figure 4).

**FIGURE 5:**
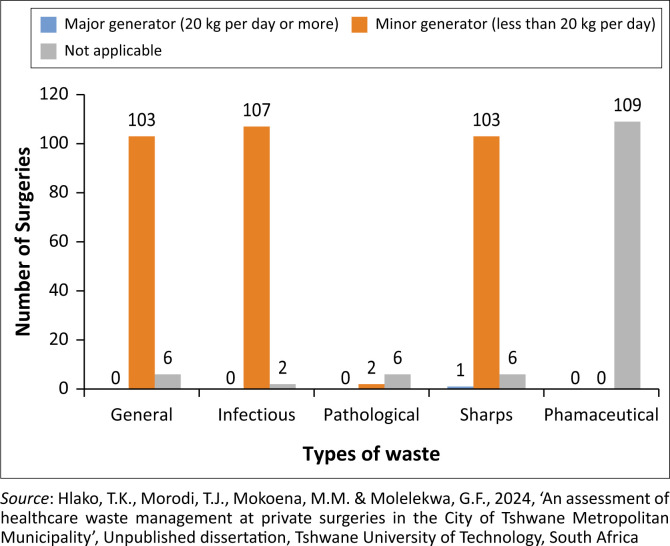
Types of healthcare waste generated by private surgeries and related generator categori.

Half of the regions each contributed at least 11 (median) private surgeries that participated in the study at a standard deviation of 10.9 private surgeries per region. Different types of HCW generated by private surgeries and related generator categories are shown in Figure 5.

The results indicate that approximately (95.7%) of private surgeries were classified as minor generators of HCW (i.e. 103 general waste, 107 infectious waste and 103 sharps waste), because they produced less than 20 kg per day. The results highlighted an important fact that private surgeries are producing more of infectious waste than general waste which has the potential to transmit diseases and cause injuries (needle pricks), therefore warrants strict management procedures and compliance to better handle hazardous HCW. Furthermore, the results revealed that 100% of the private surgeries did not generate pharmaceutical waste. This was expected because most private surgeries do not keep medication in their private surgeries. Instead, after consultation with a doctor, patients are given a prescription to go and buy medication at a pharmacy and take it home. Further analysis estimates that the maximum quantity of infectious waste that could be generated per month by the accessed private surgeries is 42 800 kg. This quantity is related to the following points, namely:

- Private surgeries are operating from Monday to Friday, which is 5 days a week.
- A total of 107 private surgeries generated infectious waste at an average of 20 kg per day.
- 20 kg per day multiply by 5 days is equals to 100 kg per week.
- 100 kg multiplied by 4 weeks per month is equals to 400 kg per month.
- 400 kg per month multiply by 107 private surgeries is equals to 42 800 kg per month.
- 42 800 kg multiply by 12 months is equals to 513 600 kg (513.6 tons).

Furthermore, all private surgeries segregated their HCW at the point of generation which demonstrated their knowledge regarding the importance of segregating waste at source (e.g. promoting and encouraging recycling of non-hazardous HCW).

The results show further that 92.7% of the generated waste was correctly stored in colour-coded containers, which signifies good practice. The demonstrated segregation of HCW at source and the usage of correct containers could be attributed to knowledge acquired through training, formal (i.e. qualifications) or informal (i.e. short courses) and legislative requirements for compliance. (i.e. Act, regulations, guidelines, norms and standards).

However, a significant majority of participants (76.1%) revealed lacking designated storage facilities for HCW as required by the *National Environmental Management Act*, 1998 (Act 107 of 1998) and section 22 of the *National Environmental Management: Waste Act*, 2008 (Act no 58 0f 2008). This led to the storage of HCW in consulting rooms prior to collection for treatment and disposal. Only 11.9% of the storage facilities used complied with the norms and standards for proper storage of hazardous HCW. This situation could lead to unauthorised access to stored hazardous HCW, thus presenting risk of infection and misuse of stored hazardous HCW by unauthorised persons.

**FIGURE 6:**
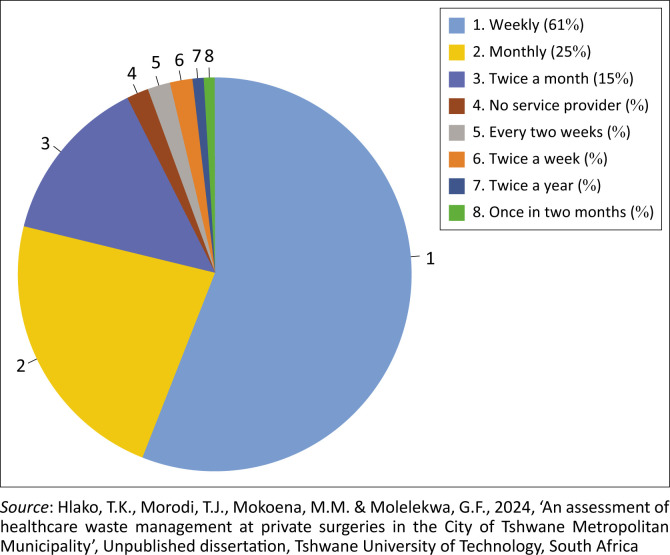
Frequencies of healthcare waste collection.

It is also noted from the study that 85% of respondents had proper personal protective equipment and 86% had appropriate hand-washing facilities, which is a positive demonstration of infection control mechanism. Finally, 92.7% of respondents indicated that their HCW was collected by private service providers (Figure 6), and confirmation thereof was by means of a waste manifest document, which was proof that the HCW was treated and disposed of at an approved facility.

**TABLE 1:** Different types of healthcare waste storage containers.

Types of HCW containers	Waste types					
	General	Infectious	Sharps	Pharmaceuticals	Radioactive	Pathological
Red container	-	-	-	-	-	Y
Cytotoxic hazard symbol container	-	-	-	Y	-	-
Radiation hazard symbol container	-	-	-	-	Y	-
Yellow bin container	-	-	Y	-	-	-
Red/yellow plastic in an infectious box	-	Y	-	-	-	-
Black and dark green container	Y	-	-	-	-	-

*Source*: South African Bureau of Standards (SABS), 1993, *SABS 0248: Code of practice for the handling and disposal of waste material within health care facilities*, South African Bureau of Standards, Pretoria

HCW, healthcare waste; Y, yes.

The results show that 61 private surgeries had their waste collected weekly, while 25 had their waste collected monthly and 15 had their waste collected twice a month. The remaining varied from once in 2 months to variable frequencies on a weekly and annual basis. Two of the private surgeries did not have any service provider for the collection of their waste, which may lead to an inability to account for the destination and of the HCW they generated. This circumstance opens the door to the illegal dumping of HCW. Thus, emphasis should be placed on HCW management education.

## Discussion

The study reveals that private surgeries generate four types of waste: general waste (32.7%), infectious waste (33.8%), pathological waste (0.635%) and sharps (32.9%). The proportion of hazardous HCW (98.7%) exceeds the global average rate of 15% reported by the WHO (2014) and Prüss et al. (2014), posing a risk of needle stick injuries, skin infections and transmission of infectious diseases such as Hepatitis A, B and C, as well as HIV. This high percentage presents potential health risks that warrant further attention that could be in a form of policy change for better planning, compliance monitoring and evaluation.

Most (95.7%) private surgeries are classified as minor generators of HCW, generating less than 20 kg per day. A significant percentage (98.2%) of participants generated infectious waste, while 94.5% generated sharps waste. The study emphasises that a large magnitude of hazardous HCW is generated annually within the South African context. According to projections by the South African Waste Information System and Recycling (Department of Environmental Affairs 2018), it was estimated that the country generates approximately 48 749 tons of hazardous HCW annually. The study emphasises about 513.6 tons of hazardous HCW could be generated annually by 109 private surgeries surveyed in the City of Tshwane Metropolitan Municipality, collectively generating significant quantities of various types of waste each month (42 800 kg). This substantial volume underscores the importance of implementing waste management strategies to mitigate health and environmental risks related to improper management of hazardous HCW which poses alarming risks of potential infections and outbreaks of diseases including emerging ones because of toxic, carcinogenic, flammable, corrosive, reactive, explosive or radioactive nature of hazardous HCW, particularly to vulnerable populations such as children who were found playing with dumped hazardous HCW in Bronkhorspruit, Region 7 of the City of Tshwane Metropolitan Municipality. Studies by Adu et al. (2020) corroborate these concerns, demonstrating that children exposed to contaminated HCW through such illegal disposal sites face increased risks of injuries (e.g., needle pricks) and subsequently contracted illnesses such as hepatitis and HIV.

This study found that private surgeries did not directly handle HCW collection, transportation, treatment or disposal services. Instead, they engaged third-party contractors for waste collection and disposal services. Most private surgeries used contracted HCW collectors at various frequency levels. For instance, 92% of participants had contracts with HCW service providers and received waste manifest documents confirming proper disposal of HCW generated in their respective private surgeries.

### Recommendations

The results of this study pointed out some gaps in the segregation and proper use of containers for temporary storage of HCW at the point of its generation. The ripple effects of improper use of hazardous HCW containers are that the entire subsequent stages of HCW management may become ineffective as incorrect waste materials might have been put inside the wrong containers, resulting in certain treatment and disposal methods not being suitable for some of the generated hazardous HCW materials (e.g. open burning and disposal of hazardous HCW together with Municipal General Waste). Thus, it is recommended that education and training workshops on the segregation of HCW, the use of colour-coded containers and the requirements for waste storage facilities be provided to all employees exposed to HCW. These workshops should be conducted annually as refresher training, in line with the guidelines set out in SANS 10248-1 (2008). Additionally, both online and in-person training programmes for healthcare workers on proper waste segregation, the use of appropriate containers and the correct temporary storage of HCW are essential to ensure safe and compliant waste management in private surgeries. Furthermore, private surgeries can use lockable mobile storage facilities, which can be effective technology for managing HCW in private surgeries, particularly in areas where high temperatures could promote microbial growth in hazardous HCW.

The study has revealed a significant gap in the full-cycle tracking of HCW quantities and quality, from generation to final disposal. Several participants did not obtain a Waste Manifest documentation from their HCW service providers, which prevented them from tracking the destination and treatment of the HCW they generated. This creates a potential risk for unlawful dumping of HCW, either by the waste generators themselves or by service providers, along the Waste management Value chain between the HCW generator, treatment facilities and landfill sites.

To address this issue, it is recommended that HCW generators must hold their service providers accountable for all waste produced and disposed of. They must demand the Waste Manifest documentation to confirm that the hazardous HCW from their facilities was properly disposed of. All the contractors that are rendering hazardous HCW disposal services to private surgeries must use a barcoded system to record the types and quantities of waste to enable traceability of HCW and accountability.

Compliance with waste management will be enforced through regular inspections by Environmental Health Practitioners (EHPs), as part of their Key Performance Areas (KPAs), specifically focusing on HCW management. Non-compliance with waste management regulations will be addressed in accordance with applicable legislation. For example:

The Constitution of the Republic of South Africa (1996) ensures that everyone has the right to a healthy environment.

Section 68 of the *National Environmental Management: Waste Act*, 2008 (Act No. 58 of 2008) stipulates penalties, including fines, for improper waste management practices in South Africa.

Section 32 of the *National Environmental Management Act*, 1998 outlines penalties for illegal waste management practices.

Therefore, this study recommends the implementation of the above measures to improve the management and monitoring of HCW from generation to final disposal.

### Limitations of the study

The study was commissioned during COVID-19 whereby movement of people and interactions were restricted. This limited the researcher’s physical interactions with the participants. Another limitation was the financial constraint which led to the reduction in the number of participants.

## Conclusion

The study assessed the practices of HCW management in the City of Tshwane Metropolitan Municipality. The study formulated four research objectives (generation, storage, treatment and disposal practices). Given the cross-cutting nature of the four objectives in practice, the results combined generation and storage, treatment and disposal practices as main headings. The results revealed that most respondents in the study were experienced medical practitioners (≥ 6 years) such as doctors and dentists, and nurses who generated HCW, most of whom (73.4%) could be classified as small generators of HCW. Additionally, they were responsible for the on-site segregation and temporary storage of the generated waste. The study found that HCW was put in different containers, which complied with the requirements for the correct containers/receptacles for HCW. However, the responses also revealed that some private surgeries did not have proper on-site storage for HCW, which excludes general waste, and they used consulting rooms, storerooms and kitchens to store HCW, which did not meet the requirements for the temporary storage of HCW, and therefore resembles a bad practice. Furthermore, the results also pointed out that the practitioners who were responsible for the private surgeries had appointed HCW service providers to ensure the proper treatment and disposal of their HCW as the general waste was collected by the City of Tshwane Metropolitan Municipality. As a result, HCW treatment and disposal were done elsewhere by HCW service provider and confirmation thereof was by means of a waste manifest document, which was proof that the HCW was treated and disposed of at an approved facility.
